# REST corepressor 2 contributes to the cell proliferation of endometrial cancer

**DOI:** 10.3389/fonc.2025.1539263

**Published:** 2025-08-27

**Authors:** Qingjuan Zhu, Xin Yang, Yuchun Lv

**Affiliations:** N19 District Gynecology, Quanzhou First Hospital, Fujian Medical University, Quanzhou, Fujian, China

**Keywords:** RCOR2, endometrial cancer, UCEC, viability, proliferation

## Abstract

**Background:**

Uterine corpus endometrial cancer (UCEC) is a prevalent gynecological malignancy. REST corepressor 2 (RCOR2), a nuclear transcription co-repressor, has been implicated in various cellular processes. However, its regulatory role in UCEC progression remains unclear.

**Methods:**

RCOR2 expression levels were analyzed in UCEC tissues and cell lines using qPCR, Western blotting. Functional assays, including CCK8 and colony formation assays, were used to assess the impact of RCOR2 knockdown or overexpression on UCEC cell viability and proliferation.

**Results:**

RCOR2 expression was significantly elevated in UCEC tissues compared to adjacent normal tissues. High RCOR2 expression correlated with advanced clinical stage, high histologic grade, and lymph node metastasis. ROC analysis indicated strong diagnostic value. RCOR2 expression showed a positive correlation with proliferation-related genes MKI67, CCND1, and PCNA. Functional assays revealed that RCOR2 knockdown suppressed, while overexpression promoted, proliferation of endometrial cancer cells. These effects were validated by CCK8 and colony formation assays, as well as changes in mRNA and protein levels of MKI67, CCND1, and PCNA, supporting RCOR2’s role in regulating UCEC cell proliferation.

**Conclusions:**

These findings suggest that RCOR2 promotes endometrial cancer progression by enhancing tumor cell proliferation and may serve as a potential diagnostic and therapeutic target in UCEC.

## Introduction

Endometrial cancer, also known as Uterine Corpus Endometrial Carcinoma (UCEC), is the most common malignancy of the female reproductive system, with a global incidence rate of approximately 4.4% (1, 2). The increasing incidence, mortality rates and 10-20% developing distant recurrence, present a significant public health challenge of UCEC (3). This highlights the urgent need for dissecting the molecular mechanisms driving UCEC and the exploring novel therapeutic methods.

One potential molecular target is RCOR2 (REST corepressor 2), a nuclear transcription co-repressor that functions primarily as a transcriptional repressor and is involved in maintaining the pluripotency of embryonic stem cells and regulating neurogenesis (4, 5). Preliminary bioinformatics analyses have indicated that RCOR2 is overexpressed in various cancers, including UCEC. This overexpression has been associated with poor prognosis in cancer patients (6, 7). Additionally, RCOR2 has been implicated in the regulation of immune responses (5, 8, 9), further suggesting its potential role in tumorigenesis and cancer progression.

Recent studies have provided insights into the mechanisms by which RCOR2 may contribute to cancer. In acute myeloid leukemia (AML), RCOR2 was found to be upregulated in B7-H4-null cells, and its knockdown resulted in reduced leukemogenesis (6, 10). This suggests that RCOR2 may promote cancer cell survival and proliferation by modulating key signaling pathways. In the context of UCEC, however, the specific functions and mechanisms of RCOR2 remain to be elucidated.

To address this gap in knowledge, this study aims to investigate the expression and functional role of RCOR2 in UCEC. Specifically, we seek to determine whether RCOR2 is upregulated in UCEC tissues and cell lines and to assess its impact on UCEC cell viability and proliferation. By investigating the expression and functional role of RCOR2 in UCEC, this study provided valuable insights of RCORs in UCEC progression.

## Materials and methods

### Patient samples

The pairs of UCEC tumor tissue samples and the adjacent tissue samples were collected from 174 patients. Written informed consent was obtained from all participants, and the study was approved by the Quanzhou First Hospital. The patients underwent surgical procedures without any chemotherapy or radiation treatment. Two independent pathologists reviewed and confirmed the diagnoses according to the standards established by the FIGO (11). Histopathological evaluation confirmed the diagnosis of UCEC in all samples. Tissues were frozen in liquid N_2_ and then stored at -80°C for the following analysis.

### Cell culture and *RCOR2* knockdown and overexpression

The UCEC cell line Ishikawa (ISK) and HEC-1A were obtained from the American Type Culture Collection (ATCC). Cells were cultured in DMEM/F12 medium (Gibco, Carlsbad, USA) supplemented with 10% fetal bovine serum (FBS) and 1% penicillin–streptomycin, and maintained at 37°C in a humidified 5% CO_2_ incubator.

For RCOR2 knockdown, siRNAs targeting RCOR2 (Catalog #EHU031001) and control siRNAs were purchased from Sigma-Aldrich (St. Louis, USA). ISK cells were seeded at 5 × 10^4^ cells per well in 6-well plates and incubated for 24 hours. Transfection was carried out using Lipofectamine 2000 (ThermoFisher Scientific, Waltham, MA) according to the manufacturer’s instructions, with a final siRNA concentration of 50 nM.

For RCOR2 overexpression, an adenovirus expressing human RCOR2 (ad-RCOR2) was obtained from Vector Biolabs (Malvern, USA). ISK cells were infected at a multiplicity of infection (MOI) of 50 in serum-free medium for 6 hours, followed by replacement with complete medium for continued culture.

### RNA extraction and gene expression analysis

Total RNA was extracted from tissue samples and cultured cells by using the Trizol (Invitrogen, Waltham, MA) according to the manufacturer’s instructions. The RNA was then reverse transcribed to cDNA using the SuperScript II RT kit (TAKARA BIO INC., Kusatsu, Japan). qPCR was performed using TaqMan Gene Expression Assays (ThermoFisher, Waltham, MA) on an ABI 7500 Real-Time PCR System (ThermoFisher, Waltham, MA). The target genes expression levels were calculated by using the 2^−ΔΔCt^ method. Primers’ sequences were listed below:

*CCND1*,
Forward: GCTGCGAAGTGGAAACCATC,
Reverse: CCTCCTTCTGCACACATTTGAA.
*MKI67*,
Forward: CTTTGGGTGCGACTTGACG,
Reverse: GTCGACCCCGCTCCTTTT.
*PCNA*,
Forward: GGCTCTAGCCTGACAAATGC,
Reverse: GCCTCCAACACCTTCTTGAG.
*GAPDH*,
Forward: GTCACCAGGGCTGCTTTTAAC,
Reverse: TGATGGGATTTCCATTGATGA.
*RCOR2*,
Forward: CACTCGCACGACAGCATGAT,
Reverse: CATCGCAATGTACTTGTCAAGC.

### Western blot

Proteins were extracted by RIPA buffer (ThermoFisher) and quantified using the BCA protein assay (Beyotime Biotechnology, Shanghai, China). Fouty µg of total protein samples were loaded and separated through the SDS-PAGE gel, and then transferred to PVDF membranes (ThermoFisher). Membranes were blocked firstly and then incubated overnight with 1^st^ antibodies that Anti-RCOR2 (1:1500, Abcam, #ab37113), Anti-β-Actin (1:2000, Abcam), Anti-Ki67 (SP6, 1:1000, Abcam, #ab16667), Anti-Cyclin D1 (SP4, 1:1000, Abcam, #ab16663), Anti-PCNA (PC10, 1:1000, Abcam).

### Cell proliferation assay

Cell proliferation was assessed using the Cell Counting Kit-8 (CCK8, C0038, Beyotime Biotechnology) and colony formation assays. Briefly, cells were seeded in 96-well plates at a density of 2×10^3^ cells per well. After transfection with si-RCOR2 or ad-RCOR2 for 24, 48, and 72 hours, CCK8 solution (10 µL) was added to wells and incubated for 2hr. Microplate reader was used for the measurement at 450 nm.

### Statistical analysis

Data were presented as mean ± standard deviation (SD). Statistical analyses were performed using GraphPad Prism 9.0. Comparisons between groups were made using Student’s *t*-test, Fisher’s exact test, Chi-square test or Brown-Forsythe ANOVA test.

## Results

### RCOR2 expression was elevated in UCEC

**Figure 1:**
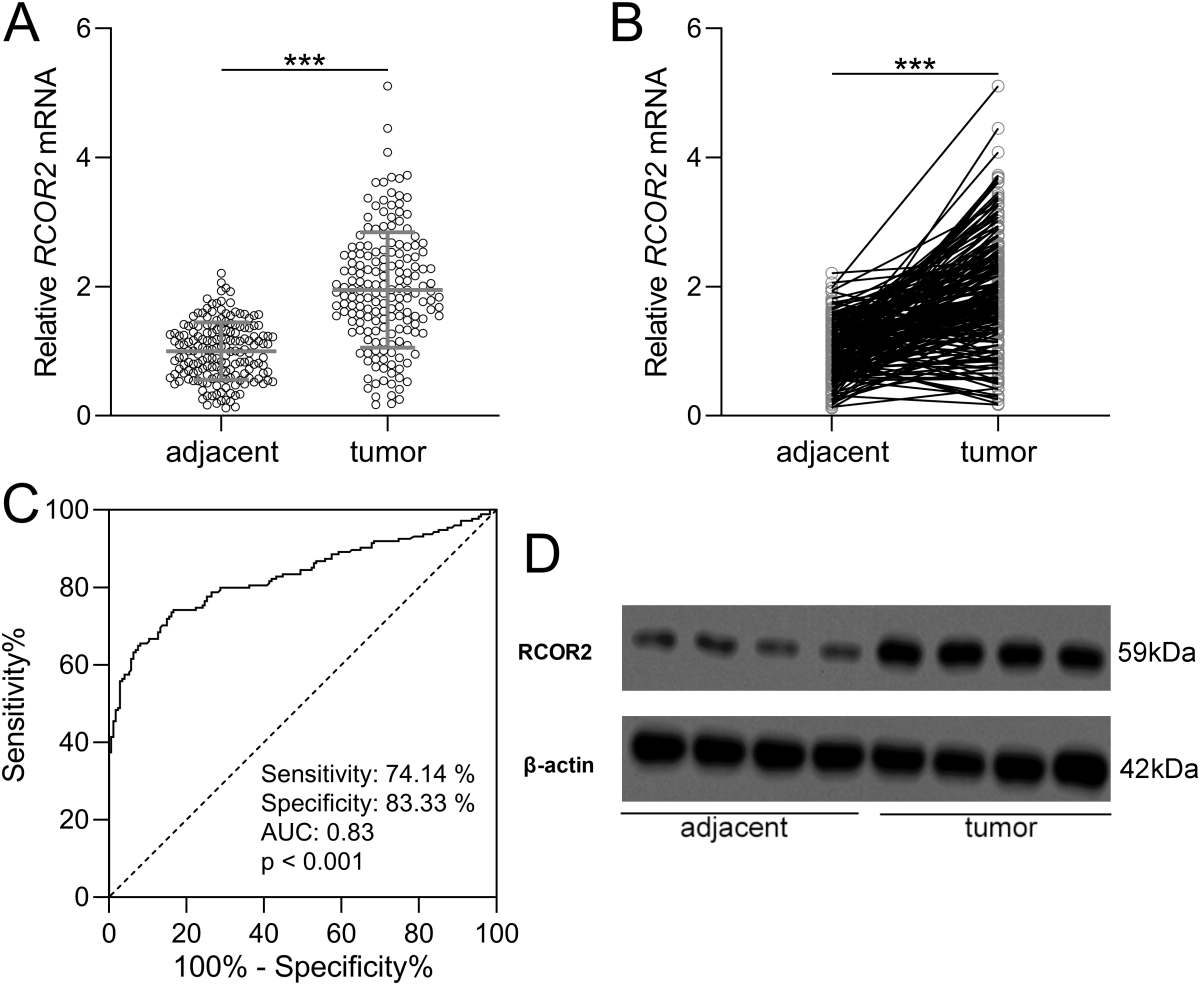
RCOR2 was overexpressed in endometrial cancer. **(A, B)** RCOR2 mRNA level in adjacent and tumor tissues of endometrial cancer patients was determined with RT-qPCR (n = 174 for each group). Data was shown with mean ± SD. ***p < 0.001 from t test. **(C)** ROC analysis of the value of RCOR2 mRNA level in diagnosis of endometrial cancer. **(D)** Representative Western blots of RCOR2 between adjacent and tumor tissues of endometrial cancer patients.

To determine the expression of RCOR2 in UCEC, qPCR and WB analyses were conducted on 174 patients’ tissue samples. Based on the mRNA expression level of RCOR2, tumor tissues were categorized into low and high expression groups (**Table 1**). The clinical and pathological characteristics of patients in the high-RCOR2 expression group were compared to those in the low-RCOR2 expression group. It was observed that RCOR2 expression was significantly associated with clinical stage, histologic grade, and lymph node metastasis (**Table 1**). The qPCR results revealed that RCOR2 expression were significantly elevated in UCEC tissues compared to adjacent control tissues (**Figures 1A, B**). ROC analysis further demonstrated the predictive value of RCOR2 expression, with sensitivity and specificity at the maximum Youden index (**Figure 1C**). Similarly, Western blot analysis confirmed the overexpression of RCOR2 protein in UCEC tissues (**Figure 1D**).

**Table 1:** *RCOR2* mRNA expression and clinicopathological factors in endometrial cancer patients (n = 174).

Variables	*RCOR2* mRNA		p value
	Low expression (n = 87)	High expression (n = 87)	
Age (years)			
< 55	37 (42.5%)	28 (32.2%)	0.209
≥ 55	50 (57.5%)	59 (67.8%)	
Clinical stage			
I-II	61 (70.1%)	43 (49.4%)	0.013
III-IV	26 (29.9%)	44 (50.6%)	
Histologic grade			
G1	37 (42.5%)	22 (25.3%)	0.034
G2	27 (31.1%)	29 (33.3%)	
G3	23 (26.4%)	36 (41.4%)	
Lymph node metastasis			
Non-metastasis	62 (71.3%)	47 (54.0%)	0.028
Metastasis	25 (28.7%)	40 (46.0%)	
Tumor invasion			
< 50%	54 (62.1%)	45 (51.7%)	0.221
≥ 50%	33 (37.9%)	42 (48.3%)	
Menopause status			
Pre	9 (10.3%)	6 (6.9%)	0.618
Peri	12 (13.8%)	10 (11.5%)	
Post	66 (75.9%)	71 (81.6%)	

Fisher’s exact test or Chi-square test.

**Figure 2:**
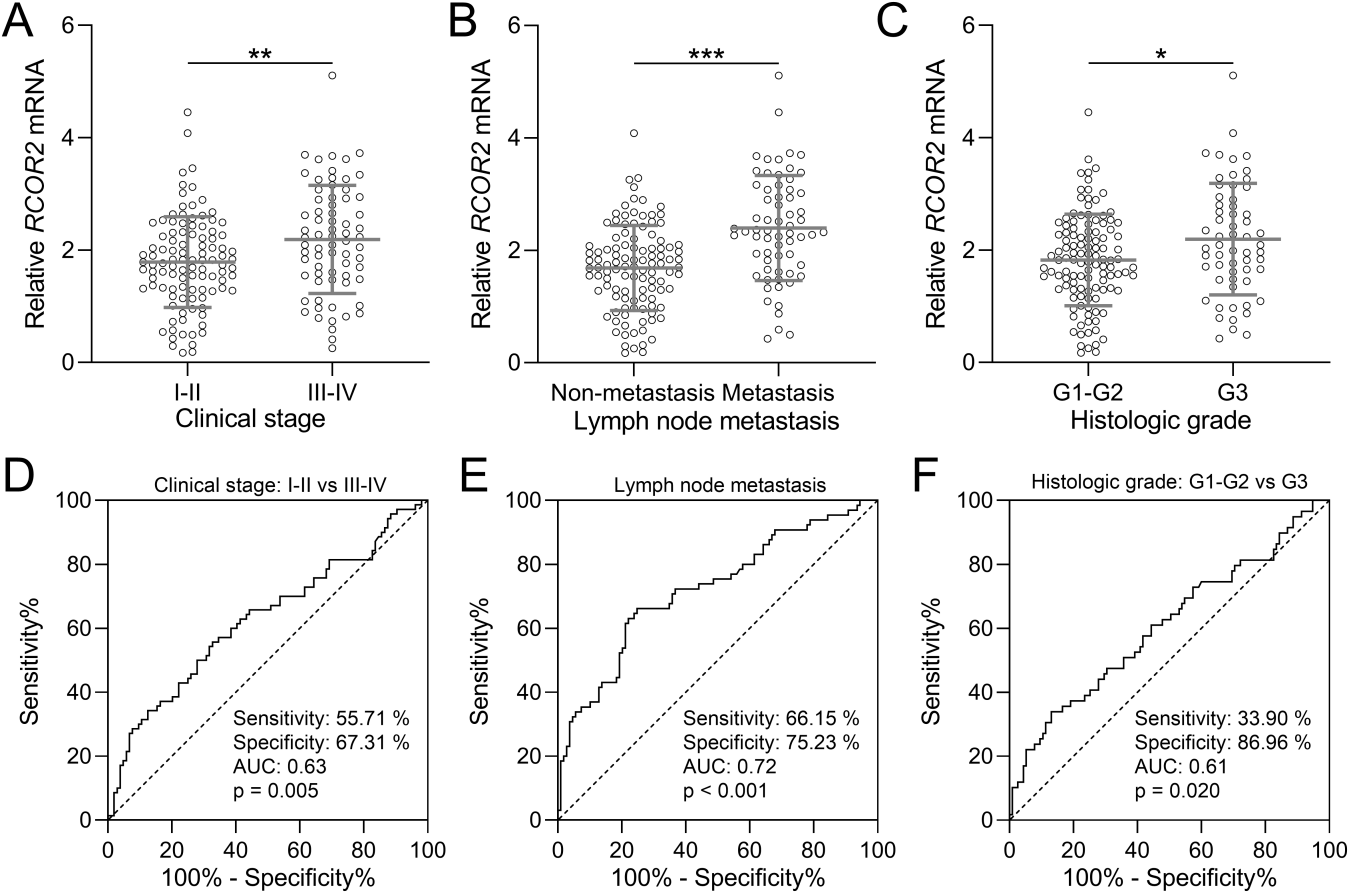
RCOR2 mRNA expression and clinicopathological factors in endometrial cancer patients. **(A)** RCOR2 mRNA expressions between clinical stage of I-II and III-IV from endometrial cancer patients (n = 104 for I-II and n = 70 for III-IV). **(D)** is the diagnostic value of RCOR2 mRNA expressions for clinical stage. **(B)** RCOR2 mRNA expressions in endometrial cancer patients without and with lymph node metastasis (n = 109 for non-metastasis and n = 65 for metastasis). **(E)** is the diagnostic value of RCOR2 mRNA expressions for lymph node metastasis. **(C)** RCOR2 mRNA expressions between histologic grade of G1-G2 and G3 from endometrial cancer patients (n = 115 for G1-G2 and n = 59 for G3). **(F)** is the diagnostic value of RCOR2 mRNA expressions for histologic grade. Data was shown with mean ± SD. *p < 0.05, **p < 0.01, ***p < 0.001 from t test.

The relationship between RCOR2 expression and clinical stage, histologic grade, and lymph node metastasis was shown in **Figure 2**. Specifically, we compared RCOR2 mRNA expression levels in tumor tissues between patients with clinical stages I-II and III-IV (**Figures 2A, D**), with and without lymph node metastasis (**Figures 2B, E**), and histologic grades G1-G2 and G3 (**Figures 2C, F**). The results show a significant correlation between RCOR2 mRNA expression and clinical stage, histologic grade, and lymph node metastasis. These findings, demonstrating significantly higher RCOR2 mRNA levels in advanced stage, metastatic, and high-grade tumors (**Figures 2A-C**), are consistent with the association of high RCOR2 expression (**Table 1**) with these adverse clinicopathological features.

### RCOR2 contributed to cell proliferation in endometrial cancer patients

**Figure 3:**
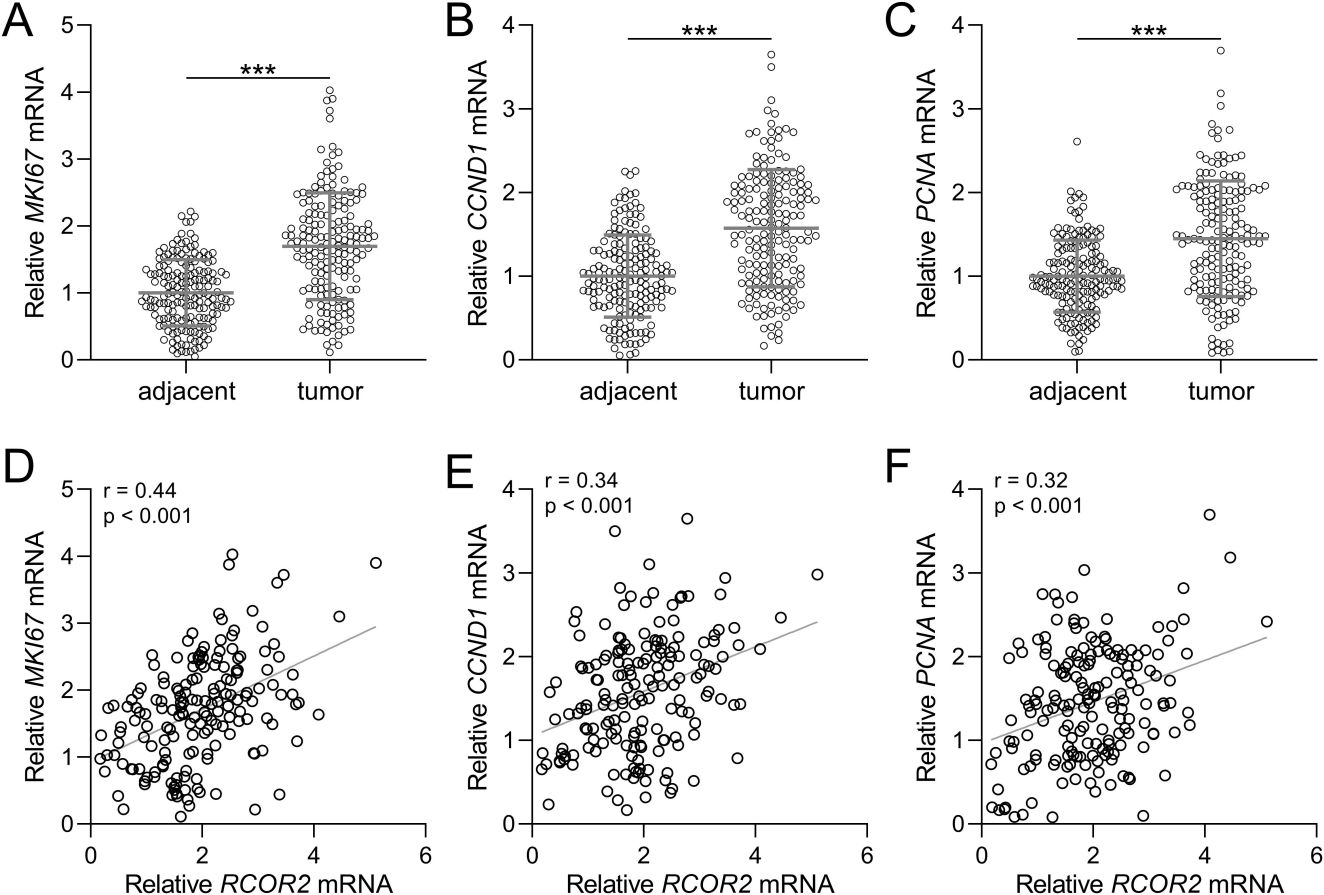
RCOR2 contributed to cell proliferation in endometrial cancer patients. **(A-C)** mRNA levels of MKI67, CCND1 and PCNA in adjacent and tumor tissues of endometrial cancer patients was determined with RT-qPCR (n = 174 for each group). Data was shown with mean ± SD. ***p < 0.001 from t test. **(D-F)** Pearson correlation coefficient analysis was employed to analyze the correlations of RCOR2 mRNA with mRNA levels of MKI67, CCND1 and PCNA in endometrial cancer patients.

Next, we analyzed the relationship between RCOR2 and the proliferation-related genes MKI67, CCND1, and PCNA under the mRNA levels, in endometrial cancer. First, the mRNA expression of all three genes were significantly elevated in tumor tissue samples compared to those in the normal tissue samples (**Figures 3A-C**). Correlation analysis showed that all three genes, MKI67, CCND1, and PCNA, exhibited a significant positive correlation with RCOR2 mRNA expression in endometrial cancer tissues (**Figures 3D-F**).

### RCOR2 regulated the proliferation of endometrial cancer cells

**Figure 4:**
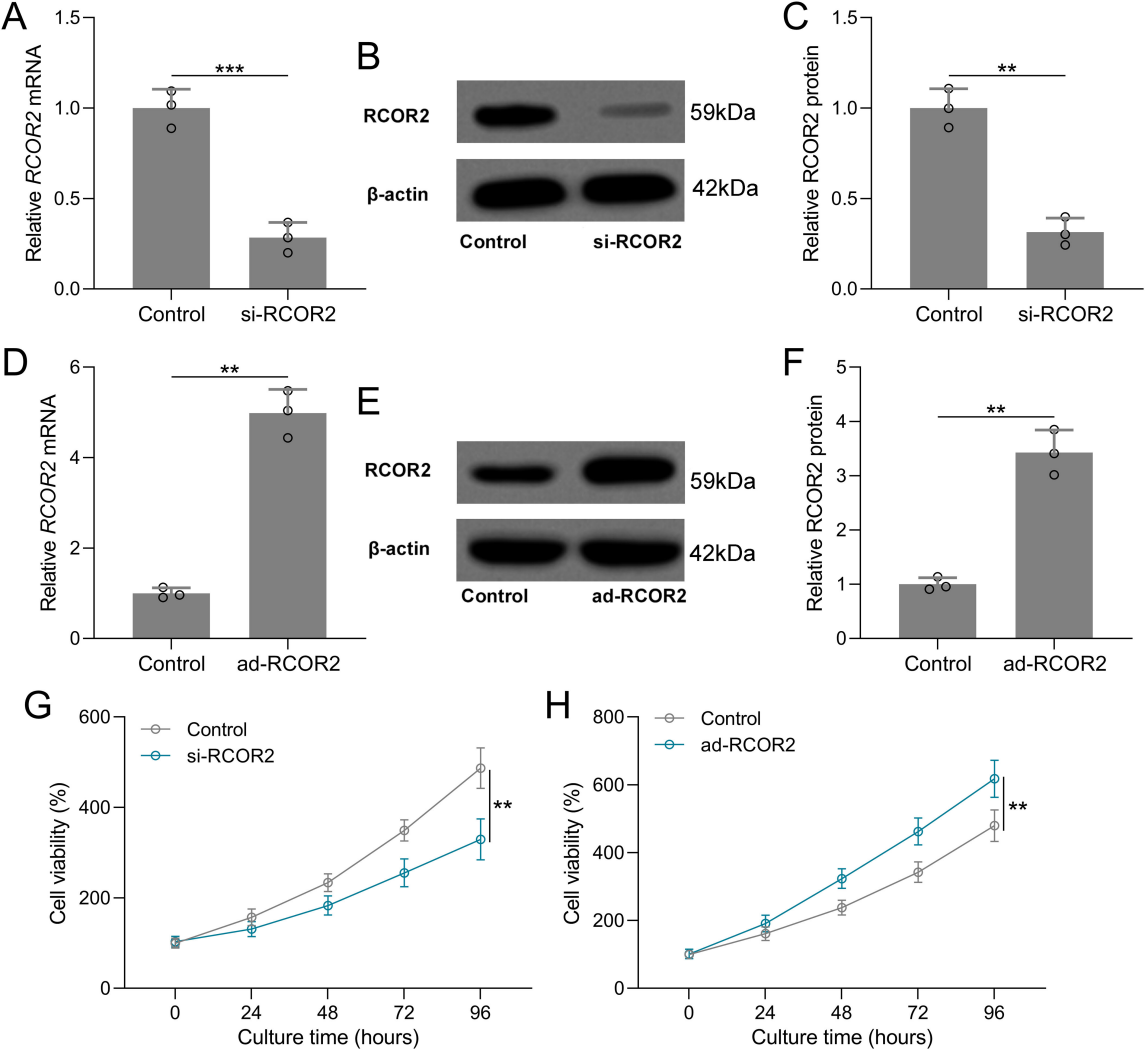
RCOR2 regulated cell viability in Ishikawa (ISK) cells. ISK cells were transfected with si-RCOR2 (50 nM) for 48 h. **(A)** qRT-PCR was used to measure the mRNA levels of RCOR2 and **(B)** representative Western blots of RCOR2. The expressions were normalized to control **(C)**. **(D)** ISK cells were transfected with ad-RCOR2 (MOI50) for 48 h. qRT-PCR was used to measure the mRNA levels of RCOR2 **(E)** and representative Western blots of RCOR2 **(F)**. ISK cells were transfected with si-RCOR2 or ad-RCOR2, cell viability was measured by CCK8 at 24, 48, 72 and 96 hours after the transfection **(G, H)**. Data was shown with mean ± SD. **p < 0.01, ***p < 0.001 from t test.

**Figure 5:**
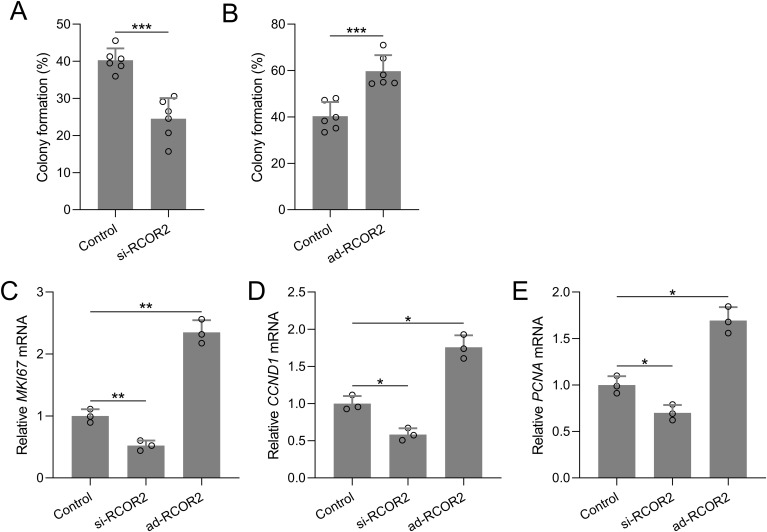
RCOR2 regulated cell proliferation in ISK cells. ISK cells were transfected with si-RCOR2 (50 nM) or ad-RCOR2 (MOI50), the colony formation assay was conducted 10 days after transfection and the formation of colonies were compared **(A, B)**. Data was shown with mean ± SD. ***p < 0.001 from t test. ISK cells were transfected with si-RCOR2 or ad-RCOR2 for 48 hours, mRNA levels of MKI67, CCND1 and PCNA were determined with RT-qPCR **(C-E)**. Data was shown with mean ± SD. *p < 0.05, **p < 0.01. Data was analyzed by Brown-Forsythe ANOVA comparing control and si-RCOR2, control and ad-RCOR2 groups.

**Figure 6:**
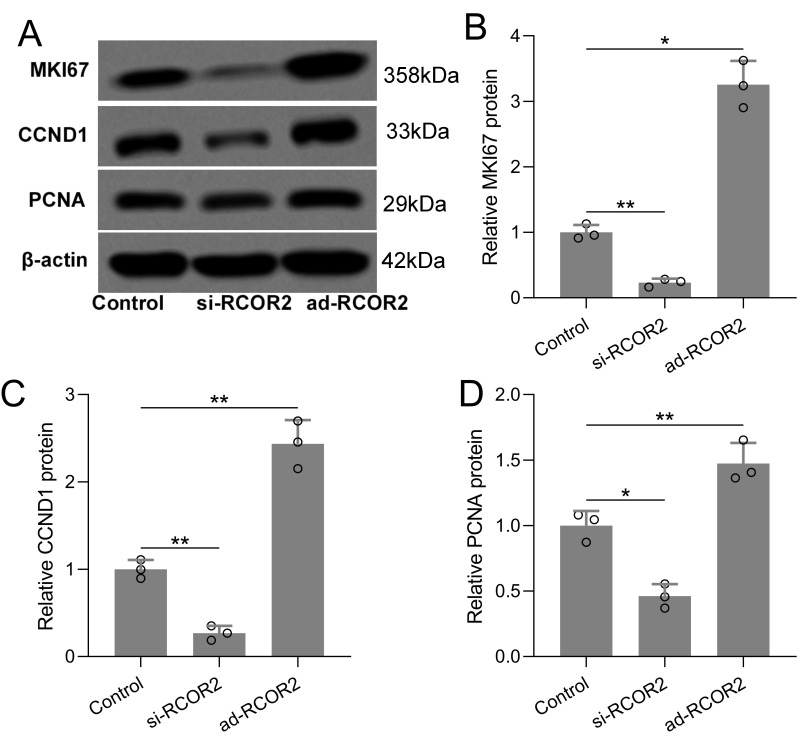
RCOR2 regulated cell proliferation in ISK cells. ISK cells were transfected with si-RCOR2 (50 nM) or ad-RCOR2 (MOI50), western blotting was used to measure the protein expressions of MKI67, CCND1 and PCNA **(A)**. The expressions were normalized to control **(B-D)**. Data was shown with mean ± SD. *p < 0.05, **p < 0.01. Data was analyzed by Brown-Forsythe ANOVA comparing control and si-RCOR2, control and ad-RCOR2 groups.

Functional assays were performed to assess the role of RCOR2 in UCEC cell behavior. RCOR2 knockdown using siRNA significantly reduced cell proliferation in ISK cells, as evidenced by the CCK8 assay (**Figures 4A-C, G**). Conversely, overexpression of RCOR2 using ad-RCOR2, could significantly enhance cell proliferation (**Figures 4D-F, H**) in ISK cells, while similar trends were identified in HEC-1A cells (**Supplementary Figures S1A, B**). The impact of RCOR2 overexpression and inhibition on endometrial cancer cell proliferation was further evaluated by using colony formation assay. The results showed that overexpression of RCOR2 in ISK cells promoted cell proliferation (**Figure 5A**), while inhibition of RCOR2 suppresses cell proliferation (**Figure 5B**). Moreover, the qPCR results of proliferation-related genes, MKI67 (**Figure 5C**), CCND1 (**Figure 5D**), and PCNA (**Figure 5E**), supported the regulatory effects of ROCR2 on endometrial cancer cell proliferation. Similar results were also found in the HEC-1A cells (**Supplementary Figures S1C, D**). Furthermore, WB showed that RCOR2 knockdown or overexpression could change the expression of MKI67, CCND1, and PCNA in endometrial cancer cells remarkedly (**Figures 6A-D**), which further demonstrated at the protein level that RCOR2 the proliferation of endometrial cancer cells.

## Discussion

This study provides the first comprehensive evidence that RCOR2 plays an oncogenic role in UCEC, particularly by regulating tumor cell proliferation, and may serve as a novel biomarker and therapeutic target. RCOR2 was significantly upregulated in UCEC tissues at both mRNA and protein levels, and its expression correlated with key clinical features including advanced stage, higher histologic grade, and lymph node metastasis, suggesting its involvement in tumor progression. These findings are consistent with emerging literature implicating transcriptional co-repressors in cancer development, such as RCOR1 and LSD1/CoREST complexes, which are known to modulate chromatin states and transcriptional programs in malignancies (6, 7, 12).

The overexpression of RCOR2 in endometrial cancer tissues and its correlation with advanced clinical stages, higher histological grades, and lymph node metastasis suggest that RCOR2 may drive tumor progression through various molecular pathways. RCOR2 has been shown to regulate the expression of genes involved in cell cycle progression, apoptosis, and differentiation, all of which are crucial processes in cancer development and progression (4, 12–14). Additionally, RCOR2 has been implicated in the regulation of immune responses, further suggesting its potential role in tumorigenesis and cancer progression (5, 8, 9).

The positive correlation between RCOR2 and proliferation markers MKI67, CCND1, and PCNA underscores its potential role in driving proliferative signaling pathways in UCEC. These genes are classical indicators of cell proliferation and have been shown to be associated with poor prognosis in multiple cancers, including breast, prostate, and cervical cancers (15–17). Functionally, RCOR2 knockdown suppressed, and overexpression promoted, UCEC cell proliferation *in vitro*, confirmed by CCK8, colony formation, and qPCR assays—supporting a causative rather than correlative role. Specifically, RCOR2 appears to regulate the cell proliferation-related genes, such as MKI67, CCND1, and PCNA, which are known markers of cell proliferation and have been implicated in the aggressive behavior of various cancers​​​​ (18–20).

The association between RCOR2 expression and poor prognosis in endometrial cancer patients is consistent with studies on other transcriptional regulators in cancer. For instance, studies have shown that downregulation of RCOR1 and RCOR2 in osteoarthritic chondrocytes contributes to increased expression of catabolic enzymes through upregulation of HES1 (12). This indicates a broader role for RCOR proteins in regulating gene expression and cellular behavior in pathological conditions.

The diagnostic value of RCOR2 was further confirmed by ROC analysis, which demonstrated strong sensitivity and specificity for distinguishing tumor aggressiveness, making RCOR2 a candidate diagnostic biomarker. This is particularly relevant given the current limitations in early detection of aggressive UCEC subtypes.

While our findings robustly demonstrate RCOR2’s role in promoting proliferation and its positive correlation with canonical proliferation markers (MKI67, CCND1, and PCNA), the underlying molecular mechanisms remain to be fully elucidated. Mechanistically, RCOR2 has been implicated in transcriptional repression networks involving REST and HDACs (21, 22), and may influence endometrial cancer progression by modulating epigenetic landscapes that control proliferation and differentiation pathways. While our findings establish a strong link between RCOR2 expression and tumor behavior, the precise molecular mechanisms by which RCOR2 modulates transcription in UCEC remain to be elucidated.

RCOR2, a member of the CoREST family of transcriptional co-repressors, is known to interact with chromatin-modifying complexes, such as LSD1 and HDACs (23), suggesting a potential role in epigenetic regulation of gene expression. It is plausible that RCOR2 promotes proliferation not only by upregulating proliferation-associated genes but also through repression of cell cycle inhibitors, such as CDKN1A (p21) or CDKN1B (p27), thereby facilitating G1/S transition (24). Additionally, RCOR2 may influence key regulatory pathways involved in cell cycle progression, including E2F and MYC signaling networks, either directly or via modulation of histone methylation and acetylation states (24, 25).

Emerging studies on epigenetic regulators in gynecologic cancers offer valuable parallels. For instance, recent work highlights the role of epigenetic remodeling in endometrial cancer proliferation and resistance, underscoring the importance of chromatin context in driving oncogenic programs (26). Similarly, findings implicate epigenetic dysregulation through histone modifiers in promoting tumor aggressiveness in breast Cancer and other gynecological cancers (27). These insights raise the possibility that RCOR2, through its association with LSD1/CoREST complexes, may contribute to the silencing of tumor suppressor genes or the activation of oncogenic enhancers.

Future investigations into RCOR2’s genomic binding sites, transcriptomic targets, and interaction partners—such as LSD1, REST, or G9a—could provide a more comprehensive understanding of its role in UCEC. Such studies may reveal whether its oncogenic role extends beyond proliferation to include pathways governing differentiation, apoptosis, or immune evasion. Exploring these mechanisms could yield novel therapeutic targets or biomarkers for aggressive subtypes of UCEC.

Although this study did not record menstrual cycle phases for premenopausal patients, their limited representation (15 of 174, 8.6%) minimizes potential impact on overall results. Over 75% of the cohort were postmenopausal, reducing variability from hormonal fluctuations. The study’s primary focus was on pathological differences between tumor and adjacent normal tissues, with RCOR2 found to be significantly overexpressed in endometrial cancer and associated with adverse clinicopathological features, including stage, grade, and lymph node metastasis. Given that tumor-related epigenetic dysregulation and abnormal cell proliferation likely exert stronger effects on RCOR2 expression than physiological hormonal changes, and considering the paired tissue design from the same patients, potential confounding from menstrual cycle phases is minimal. Thus, the key conclusion regarding RCOR2 overexpression in endometrial cancer and its clinical relevance remains robust.

Despite these promising results, several limitations exist. First, the study relied primarily on one cell line (ISK), which, while representative of UCEC, may not fully capture tumor heterogeneity. Future studies using multiple UCEC cell lines and patient-derived xenografts (PDX) or organoids would strengthen translational relevance. Second, *in vivo* validation is essential to confirm the tumorigenic role of RCOR2 and evaluate its potential as a therapeutic target.

## Conclusion

In conclusion, our study identifies RCOR2 as a key contributor to UCEC proliferation and progression, highlights its potential diagnostic utility, and opens avenues for mechanistic and therapeutic exploration. Future work should focus on elucidating the RCOR2-centered transcriptional networks and assessing the efficacy of RCOR2-targeted strategies *in vivo*.

## Data Availability

The original contributions presented in the study are included in the article/**Supplementary Material**. Further inquiries can be directed to the corresponding authors.
