# Supplementary material for: REST corepressor 2 contributes to the cell proliferation of endometrial cancer
Source: Front Oncol. 2025 Aug 27;15:1539263. doi: 10.3389/fonc.2025.1539263 (PMC12420226; doi:10.3389/fonc.2025.1539263)
Supplement: Supplementary file 1 [file DataSheet1.docx]

Supplementary materials


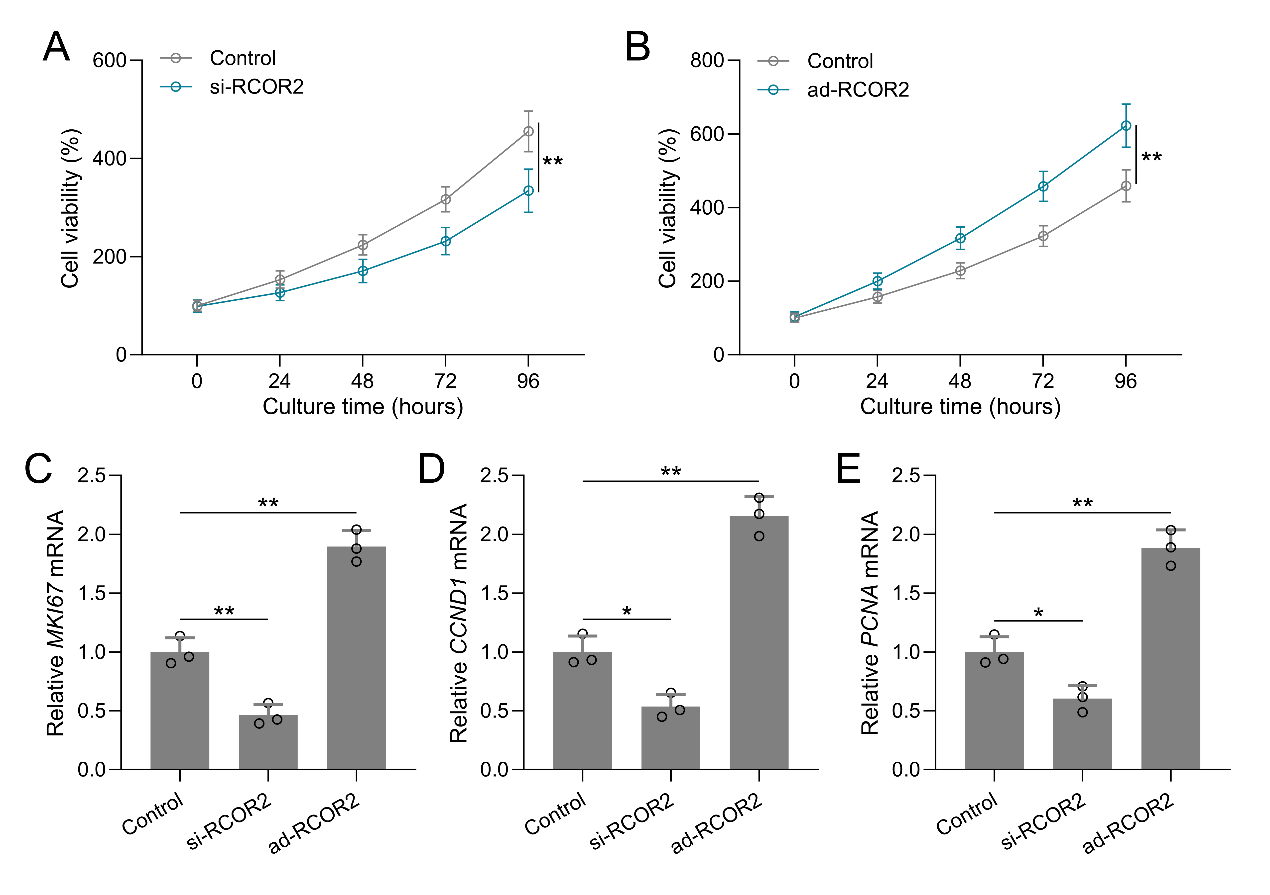


Figure S1. RCOR2 regulated cell proliferation in HEC-1A cells. HEC-1A cells were transfected with si-RCOR2 or ad-RCOR2, cell viability was measured by CCK8 at 24, 48, 72 and 96 hours after the transfection (A and B). HEC-1A cells were transfected with si-RCOR2 or ad-RCOR2 for 48 hours, mRNA levels of *MKI67, CCND1* and *PCNA* were determined with RT-qPCR (C-E). Data was shown with mean ± SD. *p < 0.05, **p < 0.01, *** p < 0.001.
